# Clinical Workflow and Substance Use Screening, Brief Intervention, and Referral to Treatment Data in the Electronic Health Records: A National Drug Abuse Treatment Clinical Trials Network Study

**DOI:** 10.5334/egems.293

**Published:** 2019-08-01

**Authors:** Li-Tzy Wu, Elizabeth H. Payne, Kimberly Roseman, Carla Kingsbury, Ashley Case, Casey Nelson, Robert Lindblad

**Affiliations:** 1 Duke University School of Medicine, US; 2 The Emmes Company, LLC, Rockville, MD, US; 3 Duke Clinical and Translational Science Institute, Duke University Medical Center, US

**Keywords:** Clinical trials network, substance use assessment, substance use screening, substance use disorder treatment

## Abstract

**Introduction:**

The use of electronic health records (EHR) data in research to inform recruitment and outcomes is considered a critical element for pragmatic studies. However, there is a lack of research on the availability of substance use disorder (SUD) treatment data in the EHR to inform research.

**Methods:**

This study recruited providers who used an EHR for patient care and whose facilities were affiliated with the National Institute on Drug Abuse’s National Drug Abuse Treatment Clinical Trials Network (NIDA CTN). Data about providers’ use of an EHR and other methods to support and document clinical tasks for Substance use screening, Brief Intervention, and Referral to Treatment (SBIRT) were collected.

**Results:**

Participants (n = 26) were from facilities across the country (South 46.2%, West 23.1%, Midwest 19.2 percent, Northeast 11.5 percent), representing 26 different health systems/facilities at various settings: primary care (30.8 percent), ambulatory other/specialty (26.9 percent), mixed setting (11.5 percent), hospital outpatient (11.5 percent), emergency department (7.7 percent), inpatient (3.8 percent), and other (7.7 percent). Validated tools were rarely used for substance use screen and SUD assessment. Structured and unstructured EHR fields were commonly used to document SBIRT. The following tasks had high proportions of using unstructured EHR fields: substance use screen, treatment exploration, brief intervention, referral, and follow-up.

**Conclusion:**

This study is the first of its kind to investigate the documentation of SBIRT in the EHR outside of unique settings (e.g., Veterans Health Administration). While results are descriptive, they emphasize the importance of developing EHR features to collect structured data for SBIRT to improve health care quality evaluation and SUD research.

## Introduction

The extent of the availability of substance use disorders (SUDs) related treatment data in the electronic health records (EHR) to support proper evaluations of the quality performance measures for SUD care and secondary use for addiction research is vastly understudied. Substance misuse and SUDs are major public health issues. The United States is amid the most catastrophic drug overdose epidemic in its history [1,2]. The drug overdose epidemic has escalated continuously for about two decades, and drug overdoses resulted in 70, 237 deaths during 2017 [2,3]. Tobacco use and alcohol use/misuse also are among the top leading preventable causes of death in the United States [4,5]. The 2017 National Survey on Drug Use and Health estimated that 22.4 percent of Americans aged 12 years or older (representing 61.1 million) were current tobacco users and that approximately 24.5 percent Americans aged 12 or older (representing 66.6 million) were binge alcohol users (i.e., drinking five or more drinks for males or four or more drinks for females on the same occasion on at least day in the past 30 days) [6]. Overall, an estimated 7.5 percent of Americans aged 12 years or older had alcohol or drug use disorder in the past year [6]. Despite the benefits of SUD treatment, the majority of Americans with SUD have not received adequate SUD treatment [7,8]. It was estimated that only 7.6 percent of adults with alcohol or drug use disorder in the past year received substance misuse related treatment at a specialty facility (e.g., drug or alcohol rehabilitation facilities, hospital inpatient services, or mental health centers) in the past year [9].

The economic costs of substance misuse and SUDs, including the costs related to lost work productivity, crime, and health care utilization, were estimated to be over $740 billion annually [10,11,12]. The already staggering costs, however, increase considerably over time. As shown from the national opioid epidemic [13], the cost of not implementing a workable health care system to provide timely prevention and treatment for SUDs is simply too high. Opioid use disorder in the United States was the 11^th^ leading cause of disability in 1990, and it was the 7^th^ leading cause in 2016 [14]. A report showed that economic costs of the opioid crisis was estimated to be $504.0 billion in 2015, which was about six times greater than a previous estimate [13]. Therefore, the implementation of Substance use screening, Brief Intervention, and Referral to Treatment (SBIRT) services to improve early detection of substance misuse and identification for SUDs for intervention and treatment at medical settings is one priority area of health care reforms [15,16,17]. The continued rise in the rates of drug overdose deaths demonstrate a clear need to study the availability of SUD related treatment data (i.e., SBIRT data) in the EHR in order to inform patients’ quality of health care for SUDs and secondary use of SUD treatment data for addiction research, such as pragmatic trials [2,18,19].

Specifically, the Health Information Technology for Economic and Clinical Health (HITECH) Act of 2009 mandated the adoption and implementation of the EHR system and its meaningful use by health care systems and organizations to improve the efficiency and quality of health care they provide [20]. In addition, the Mental Health Parity and Addiction Equity Act of 2008 required commercial health plans that offer coverage to manage benefits for behavioral health services (e.g., SBIRT for SUDs) the same as they manage other medical conditions [21]. The Affordable Care Act of 2010 further extended health insurance benefits to millions of previously uninsured Americans, including persons with SUD [17]. These health policy reforms were estimated to expand behavioral health benefits and parity protections for over 60 million Americans [22]. The health care reforms offer opportunities to improve care for Americans with SUD by promoting integration of SUD treatment and mainstream health care through the implementation of SBIRT services in medical settings. Thus, the meaningful use of the EHR and documentation of SBIRT information in the EHR are becoming an integral part of health care quality improvement for SUD care.

To properly evaluate behavioral health care quality for patients with SUD and the progress of health care reform efforts (e.g., value-based payment program), high-quality SUD related treatment information that accurately represents health care provided to patients must be available in the EHR [23,24]. A number of organizations have developed or endorsed health care measures to facilitate evaluations of the delivery and quality of patient care in order to inform performance improvement for behavioral health care services (e.g., SBIRT services). For example, the National Committee for Quality Assurance (NCQA) has developed the Healthcare Effectiveness Data and Information Set (HEDIS) quality measures aimed at improving the health and well-being of people. HEDIS is one of health care’s most widely used performance improvement tools [25]. HEDIS measures for SUD treatment services include: (a) unhealthy alcohol use screening and follow-up; (b) identification of alcohol and other drug services; (c) initiation and engagement of alcohol and other drug abuse or dependence treatment; (d) medical assistance with smoking and tobacco use cessation; and (e) follow-up after emergency department visit for alcohol and other drug abuse or dependence. Similarly, the National Quality Forum (NQF) has endorsed behavioral health measures for improving the delivery of behavioral health services for SUDs and patient outcomes of the U.S. population, which includes measures for tobacco use screening and treatment, alcohol use screening and brief intervention, and alcohol and drug use disorder treatment [26,27]. All such quality measures rely on the use of the EHR data to evaluate the quality of SUD care (i.e., SBIRT).

The EHR data also open opportunities for a paradigm shift in clinical research, including safety surveillance research, comparative effectiveness studies, pragmatic randomized trials, and registry-based randomized studies [28,29]. The longitudinally aggregated EHR data from large, diverse populations at real-word settings are considered key elements for evidence development within the framework of developing a continuously learning health system [30,31]. Pragmatic randomized trials enriched with routinely collected EHR data are considered to have the potential to be less costly and produce more generalizable results (e.g., a large sample size, efficient recruitment from the patient populations, and longer follow-up time) than traditional randomized trials [29,32]. A recent review found that the use of the EHR data for clinical trial recruitment was an effective method due to its capability for patient identification outside working hours and fast processing time [33]. However, the completeness and accuracy of the EHR data must be evaluated and addressed in order to fully realize the potential of leveraging the EHR data for health care quality improvement and research efforts [29,32,33]. For example, a study analyzing 351 eligibility criteria from 15 clinical trials found that clinical data was available for 64 percent of all patients and that the total completeness of EHR data for recruitment purposes was just 35 percent [34].

The use of the EHR data for clinical trials and observational studies have increased considerably in the past decade [28]. Despite the fact that the drug overdose death crisis has been escalating for about two decades in the United States [2,3], there is a scarcity of empirical data on the availability substance use screening and SUD treatment data in the EHR. Prior findings from qualitative studies conducted in the U.S. Veterans Health Administration setting have suggested a pattern of inconsistent implementation and documentation of substance use screening and brief intervention [35,36,37]. The ongoing national opioid epidemic indicates an urgent need to study the quality of SUD treatment data in the EHR. In April 2018, the National Institutes of Health launched the HEAL (Helping to End Addiction Long-term^SM^) Initiative, an aggressive, trans-agency effort to speed scientific solutions to stem the national opioid public health crisis [1,38]. Primary research areas for the HEAL Initiative are to identify effective strategies and health care models to improve prevention and treatment for opioid misuse and addiction and to enhance pain management while reducing substance misuse [39]. The HEAL Initiative not only is expected to increase the number of observational studies and clinical trials that includes the EHR data for research, but also promote the use of the EHR data to evaluate the uptake and quality of SUD treatment. Thus, there is a pressing need for research on the completeness of the SBIRT data in the EHR.

To fill the gap for a lack of research data on the availability of the SUD treatment in the EHR, the goal of this study is to use an evidence-based SBIRT approach as a framework in order to understand the clinical workflow and documentation of the SUD related treatment data by SBIRT clinical tasks in the EHR. SBIRT is a practical framework for intervening spectrum of substance use and SUD by enabling providers to quickly assess the level of substance misuse to determine the treatment needs and provide treatment services [15]. The key steps the SBIRT includes the use of screeners to identify substance users, assessment for levels of substance misuse/SUDs using validated tools/instruments among those with positive screening results, and provision of brief intervention (e.g., counseling, motivational interviewing) and/or treatment (e.g., pharmacotherapy, referral to a specialist) based on the patient’s health care needs [15,16]. These steps of screening substance use, assessing the persistence of SUD, conducting brief intervention, providing treatment for SUD, or making a referral to a specialist reflect the quality measures – process and outcome measures – for SUD’s preventive and treatment services endorsed by NCQA, NQF, and the Joint Commission [25,26,27]. This SBIRT workflow also is consistent with the U.S. Preventive Services Task Force’s recommendations for screening and intervening tobacco use and unhealthy alcohol use in medical settings [40,41].

This study is conducted within the National Institute on Drug Abuse’s National Drug Abuse Treatment Clinical Trials Network (NIDA CTN). The NIDA CTN currently includes 13 research nodes (networks) across the nation that provide a means to conduct multisite clinical trials of SUD related conditions to determine intervention effectiveness across settings and diverse populations, with the goal of transferring study results to providers and patients. Like researchers from other medical fields [28], there is a growing interest in utilizing the EHR data for addiction research within the NIDA CTN [42,43]. In particular, the optimal goal for conducting multisite pragmatic addiction treatment trials within a research network, such as NIDA CTN, is to generate “real-world evidence” that will be useful to inform feasible health care strategies for prevention or treatment for SUDs. The challenges facing the integration of the EHR data with research data to support clinical trials must be studied and addressed in order to produce valid findings to inform clinical practice for SUD care. This study leverages the health systems and facilities affiliated with the NIDA CTN to explore the clinical workflow of SBIRT and documentation of the SUD treatment based on the SBIRT clinical tasks in the EHRs. The results will help inform the availability of the SUD treatment in the EHR and identify areas of challenges (e.g., clinical workflow) that should be addressed or researched.

## Methods

### Study sample

The target sample included providers (e.g., Physician, Nurse Practitioner, Registered Nurse, Physician Assistant, Clinical Psychologist) using an EHR for patient care and working at a facility affiliated with the NIDA CTN. It was expected that approximately 3–4 providers from different practices or facilities would be recruited from each CTN node. To increase the diversity of the EHR system and practice settings, each participant was recruited from different clinical settings/facilities.

### Data collection

Before the study initiation, research coordinators completed Human Subject Research, Good Clinical Practice, and protocol-specific training (including participating in a pilot interview with one addiction provider and one non-addiction provider). Research coordinators contacted CTN node coordinators at all 13 nodes to obtain a list of potential participants from different facilities or clinical settings. Research coordinators then emailed potential participants the IRB-approved information to introduce the study, which was followed by making phone calls to recruit participants and obtaining the verbal consent by phone. A waiver of documentation of consent was approved by Duke University Health System Institutional Review Board.

**Figure 1:**
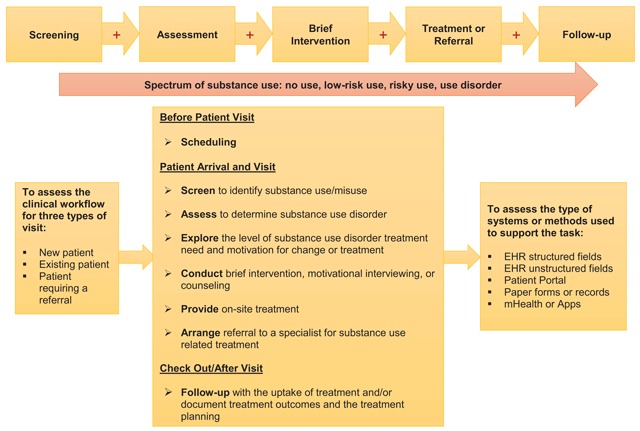
The clinical workflow of substance use screening, brief intervention, and referral to treatment.

Participants’ demographics and facility/specialty were collected in a brief questionnaire that included discrete response categories for questionnaire items. Participants’ facility location was used to define a variable indicating the U.S. census-defined region (South, West, Midwest, and Northeast). To properly identify clinical tasks and associated EHR data for SUD treatment services – process and outcome related quality measures endorsed by NCQA and NQF – that represent evidence-based SUD treatment services provided by a provider [25,26,27,40,41], an SBIRT workflow was used to guide the clinical workflow interview of the SBIRT tasks [15,44,45]. Figure 1 summarizes the framework for conducting the SBIRT workflow interview. SBIRT is an evidence-based practice model used to increase early detection of substance misuse and SUD, promote SUD prevention and treatment, and reduce adverse consequences from substance misuse and addiction [44]. SBIRT has been promoted by the Substance Abuse and Mental Health Services Administration of the U.S. Department of Health and Human Services as the primary model to facilitate the integration of the behavioral health care (e.g., SUD prevention and treatment services) into health care settings [44]. The use of the SBIRT workflow helped to reduce respondent bias by standardizing the information collected for SUD related clinical tasks,

To understand the completeness and availability of the SBIRT related data in the EHR, the clinical workflow interview asked the participant about his/her use of the EHR and other methods, including patient portal, paper form, and mHealth/app, in conducting patient care and documenting SBIRT related activities. To control for patient’s visit type, the workflow was explored for three visit types: new patient, existing patient, and patient requiring a referral. Based on the clinical workflow assessment tool, the clinical tasks were organized into the following main steps: before patient visit (scheduling), patient arrival and visit, and check out/after visit [46]. These steps allowed an evaluation of clinical tasks and associated EHR information for the processes and outcomes of the SBIRT services [25,26,27,40,41].

For *“Before Study Visit,”* research staff asked whether the provider requested the patient to complete health risk screen form(s) while scheduling a patient visit, including substances screened, tools/forms used to screen substance use, and types of systems used to support and document the workflow (e.g., where the information was documented). For *“Patient Arrival and Visit,”* research staff assessed whether the participant (a) *screened* the patient to identify substance use or misuse upon patient arrival for his/her visit, (b) assessed the patient to determine the risk of having SUD or to confirm it, (c) explored the level of SUD treatment need or motivation for change or SUD treatment options, (d) conducted brief intervention, counseling, or other psychotherapy for problem substance use or SUD, (e) provided on-site treatment (e.g., prescribing medications for nicotine dependence, alcohol use disorder, opioid use disorder), and (f) arranged a referral to a specialist for SUD related treatment (e.g., addiction or mental health facility). For *“Check Out/After Visit,”* research staff assessed whether the participant conducted the follow-up to know the uptake of the specialty treatment, documented the follow-up treatment or outcomes, or conducted other after-visit services.

To collect key data elements from the clinical workflow of SBIRT clinical tasks and reduce errors in coding open-ended response, each of clinical workflow questions included discrete response categories for each task (i.e., yes, no, don’t know), a list of substances intervened/discussed for the task, and a list of types of systems used for documenting/supporting the task (e.g., where the information was documented). Comment boxes also were used for each workflow question to capture additional information to clarify participant’s response, including tools/forms/measures used to screen substance use, assess the SUD diagnosis, and explore SUD treatment needs; information of brief intervention or psychotherapy provided by the provider, medications prescribed for a SUD; and other follow-up services conducted by the provider. The use of an interview method was considered to collect more comprehensive information about clinical workflow processes than having the provider fill out the questionnaire online or via a mailed version. The interview took approximately 2 hours to complete. Each participant received $200 for study compensation. The study was conducted between December 2016 and June 2017.

### Data analysis

The study dataset was managed and analyzed by NIDA CTN’s Data and Statistics Center. Descriptive analysis was conducted to summarize results regarding the use of an EHR or other systems to support clinical tasks and documentation of SBIRT information. For continuous variables, descriptive statistics included number of observations, mean, and standard deviation. Discrete variable summaries included counts, proportions, and/or frequencies and percentages as appropriate. To explore differences between addiction providers and non-addiction providers in performing key SBIRT tasks, exact tests for proportions were conducted.

## Results

### Demographic and facility characteristics

A total of 32 individuals from 12 CTN nodes (networks) were identified and contacted by email to introduce the study. All individuals accepted the study invitation. Three did not complete the interview due to the scheduling difficulty for the phone interview. Three participants were non-providers. This analysis focused on 26 provider participants who completed the interview.

As shown in Table 1, the mean age of participants was 45.7 years (range: 33–65 years). Of the sample (n = 26), 65.4 percent were female and 76.9 percent were white. The 26 participants were from 11 CTN nodes (South 46.2 percent, West 23.1 percent, Midwest 19.2 percent, Northeast 11.5 percent). About two thirds (65.4 percent) had a professional degree (e.g., M.D.). The majority of participants (n = 23, 88.5 percent) were from large health systems (n = 19, academic-settings; n = 4, non-academic settings). Another three providers were from three independent practices. Participants were recruited from various settings: ambulatory primary care (30.8 percent), ambulatory other/specialty (26.9 percent), mixed setting (partial hospitalization in day/night clinic, 11.5 percent), hospital outpatient (11.5 percent), inpatient (3.8 percent), emergency department (7.7 percent), and other (7.7 percent). Most participants practiced either addiction (38.5 percent) or family/internal (38.5 percent) medicine; additionally, 23.0 percent provided care for non-addiction mental health, pediatrics, emergency medicine, or infectious disease.

**Table 1:** Demographics and clinical characteristics.

Study Sample	n = 26

**Age**	
Mean (SD)	45.7 (9.15)
Median (Min, Max)	43.5 (33.0, 65.0)
**Sex – n (%)**	
Male	9 (34.6%)
Female	17 (65.4%)
**Race – n (%)**	
White	20 (76.9%)
Black or African American	1 (3.8%)
Asian	2 (7.7%)
Multiple Races	2 (7.7%)
Not Reported	1 (3.8%)
**Ethnicity – n (%)**	
Non-Hispanic/Latino	25 (96.2%)
Hispanic/Latino	1 (3.8%)
**Education level – n (%)**	
Associate or master degree	4 (15.4%)
Professional school degree (e.g., MD)	17 (65.4%)
Doctoral degree (e.g., PhD, EdD)	5 (19.2%)
**Facility state region – n (%)**	
West	6 (23.1%)
South	12 (46.2%)
Midwest	5 (19.2%)
Northeast	3 (11.5%)
**Facility part of larger group/health system – n (%)**	
No	3 (11.5%)
Yes	23 (88.5%)
**Practice specialty^2^ – n (%)**	
Inpatient	1 (3.8%)
Emergency department	2 (7.7%)
Hospital outpatient	3 (11.5%)
Ambulatory primary care	8 (30.8%)
Ambulatory other/specialty	7 (26.9%)
Mixed setting (e.g., partial hospitalization in day/night clinic)	3 (11.5%)
Other	2 (7.7%)
**Type of care provided^2^ – n (%)**	
Addiction specialty	10 (38.5%)
Mental health (non-addiction)	2 (7.7%)
Family or internal medicine	10 (38.5%)
Pediatrics	1 (3.8%)
Emergency medicine	1 (3.8%)
Other (infectious disease, pediatrics/emergency medicine)	2 (7.7%)

^1^ The study sample included providers considered to have completed the interview, based on identified criteria. ^2^ Specified and ‘Other’ categorizations were added from free text responses.

### Clinical workflow of SBIRT services

Table 2 summarizes results of SBIRT related clinical tasks for each visit type both overall and by addiction or non-addiction provider specialty.

**Table 2:** Clinical workflow for substance use screening, brief intervention, referral to treatment for substance use disorder, and follow-up for update of treatment.

Visit Type	New Patient			Existing Patient			Patient Requiring a Referral		

Provider’s Specialty	Total (n = 26)	Addiction specialty (n = 10)	Non-addiction specialty (n = 16)	Total (n = 26)	Addiction specialty (n = 10)	Non-addiction specialty (n = 16)	Total (n = 26)	Addiction specialty (n = 10)	Non-addiction specialty (n = 16)

Provider’s positive response	n (%)	n (%)	n (%)	n (%)	n (%)	n (%)	n (%)	n (%)	n (%)

**Before patient visit**									
Requested health risk screen while scheduling	5 (19.2%)	2 (20.0%)	3 (18.8%)	0	0	0	0	0	0
**Patient arrival and visit**									
Screened for substance use	25 (96.2%)	10 (100.0%)	15 (93.8%)	21 (80.8%)	7 (70.0%)	14 (87.5%)	17 (65.4%)	6 (60.0%)	11 (68.8%)
Assessed for substance use disorders	22 (84.6%)	9 (90.0%)	13 (81.3%)	20 (76.9%)	9 (90.0%)	11 (68.8%)	11 (42.3%)	4 (40.0%)	7 (43.8%)
Explored treatment needs and options	26 (100.0%)	10 (100.0%)	16 (100.0%)	25 (96.2%)	10 (100.0%)	15 (93.8%)	22 (84.6%)	8 (80.0%)	14 (87.5%)
Conducted brief intervention or counseling	24 (92.3%)	10 (100.0%)	14 (87.5%)	24 (92.3%)	10 (100.0%)	14 (87.5%)	21 (80.8%)	7 (70.0%)	14 (87.5%)
Provided on-site treatment for substance use disorder(s)	21 (80.8%)	8 (80.0%)	13 (81.3%)	21 (80.8%)	8 (80.0%)	13 (81.3%)	14 (53.8%)	5 (50.0%)	9 (56.3%)
Arranged the referral to a specialist	23 (88.5%)	7 (70.0%)*	16 (100.0%)*	24 (92.3%)	9 (90.0%)	15 (93.8%)	23 (88.5%)	8 (80.0%)	15 (93.8%)
**Check out and after visit**									
Followed up with the uptake of specialty treatment	20 (76.9%)	7 (70.0%)	13 (81.3%)	24 (92.3%)	9 (90.0%)	15 (93.8%)	23 (88.5%)	9 (90.0%)	14 (87.5%)
Conducted other after-care services or follow-ups	8 (30.8%)	2 (20.0%)	6 (37.5%)	9 (34.6%)	2 (20.0%)	7 (43.8%)	4 (15.4%)	1 (10.0%)	3 (18.8%)

* Exact test: P-value = 0.046 for comparing the proportion of addiction and non-addiction specialists who would arrange the referral to a specialist for new patients.

**New patient:** Just 19.2 percent of the sample reported screening the new patient for health risk (e.g., substance use) while scheduling the visit. Upon patient arrival and visit, the majority of the sample would screen the patient for substance use (96.2 percent), assess SUD (84.6 percent), explore treatment needs/options (100 percent), conduct brief intervention/counseling (92.3 percent), provide on-site treatment for SUD (80.8 percent), arrange the referral to a specialist (88.5 percent), or follow up with the uptake of specialty treatment at the visit checkout (76.9 percent).

**Existing patient:** None reported screening the existing patient for health risk while scheduling the visit. After patient arrival and visit, the majority of the sample would screen the patient for substance use (80.8 percent), assess SUD (76.9 percent), explore treatment needs/options (96.2 percent), conduct brief intervention/counseling (92.3 percent), provide on-site treatment for SUD (80.8 percent), arrange the referral to a specialist (92.3 percent), or follow up with the uptake of specialty treatment at the visit checkout (92.3 percent).

**Patient requiring a referral:** None reported screening the patient requiring a referral for health risk while scheduling the visit. After patient arrival and visit, a high proportion of the sample would screen the patient for substance use (65.4 percent), assess SUD (43.2 percent), explore treatment motivation/options (84.6 percent), conduct brief intervention/counseling (80.8 percent), provide on-site treatment for SUD (53.8 percent), arrange the referral to a specialist (88.5 percent), or follow up with the uptake of specialty treatment at the visit checkout (88.5 percent).

**Provider specialty:** A total of 10 participants were addiction specialists, while 16 participants practiced other types of non-addiction care. Exploratory exact tests were conducted to explore differences in performing SBIRT tasks by addiction provider status (yes vs. no). The proportion of participants who would arrange the referral to a specialist for a new patient differed significantly between addiction providers and non-addiction providers (70.0 percent vs. 100 percent, P = 0.046). Other tasks did not differ statistically by addiction provider status.

**Substances screened and tools used:** While SBIRT services were performed, clinical tasks for substance use screen, SUD assessment, treatment option exploration, provision of on-site treatment, referral to a specialist, and follow-up of the uptake of SUD treatment were performed for multiple substances (tobacco, alcohol, and drugs). The pattern was similar across different types of visit. Validated tools were rarely used for substance use screen, SUD assessment, and treatment option exploration.

### Documentation of SBIRT information

Table 3 summarizes the use of the EHR and other methods to document SBIRT data by visit type. The results are based on the subsamples of providers who responded positively to the SBIRT task.

**Table 3:** The electronic health records (EHR) and other methods used by providers to document clinical tasks (n = 26).

Workflow and clinical tasks – n (%)	New Patient	Existing Patient	Patient Requiring a Referral

**Requested health risk screen while scheduling, yes**	**5 (19.2%)**	**0 (0%)**	**0 (0%)**
**Systems or methods used**	**n = 5**	**n = 0**	**n = 0**
EHR structured fields	2 (40.0%)	… … ..	… … ..
EHR unstructured fields (e.g., progress notes)	3 (60.0%)	… … ..	… … ..
Patient Portal	2 (40.0%)	… … ..	… … ..
Paper forms or records	2 (40.0%)	… … ..	… … ..
mHealth or apps	… … ..	… … ..	… … ..
**Screened substance us, yes**	**25 (96.2%)**	**21 (80.8%)**	**17 (65.4%)**
**Systems or methods used**	**n = 25**	**n = 21**	**n = 17**
EHR structured fields	20 (80.0%)	10 (47.6%)	9 (52.9%)
EHR unstructured fields (e.g., progress notes)	19 (76.0%)	18 (85.7%)	15 (88.2%)
Patient Portal	5 (20.0%)	3 (14.3%)	3 (17.6%)
Paper forms or records	6 (24.0%)	5 (23.8%)	5 (29.4%)
mHealth or apps	1 (4.0%)	1 (4.8%)	1 (5.9%)
**Assessed substance use disorder, yes**	**22 (84.6%)**	**20 (76.9%)**	**11 (42.3%)**
**Systems or methods used**	**n = 22**	**n = 20**	**n = 11**
EHR structured fields	17 (77.3%)	16 (80.0%)	9 (81.8%)
EHR unstructured fields (e.g., progress notes)	13 (59.1%)	9 (45.0%)	3 (27.3%)
Patient Portal	2 (9.1%)	3 (15.0%)	2 (18.2%)
Paper forms or records	3 (13.6%)	1 (5.0%)	… … ..
mHealth or apps	… … ..	… … ..	… … ..
**Explored treatment motivation or options, yes**	**26 (100.0%)**	**25 (96.2%)**	**22 (84.6%)**
**Systems or methods used**	**n = 26**	**n = 25**	**n = 22**
EHR structured fields	7 (26.9%)	5 (20.0%)	2 (9.1%)
EHR unstructured fields (e.g., progress notes)	25 (96.2%)	24 (96.0%)	21 (95.5%)
Patient Portal	2 (7.7%)	3 (12.0%)	3 (13.6%)
Paper forms or records	2 (7.7%)	1 (4.0%)	… … ..
mHealth or apps	… … ..	… … ..	… … ..
**Conducted brief intervention or counseling, yes**	**24 (92.3%)**	**24 (92.3%)**	**21 (80.8%)**
**Systems or methods used**	**n = 24**	**n = 24**	**n = 21**
EHR structured fields	4 (16.7%)	3 (12.5%)	1 (4.8%)
EHR unstructured fields (e.g., progress notes)	24 (100.0%)	24 (100.0%)	21 (100.0%)
Patient Portal	3 (12.5%)	3 (12.5%)	3 (14.3%)
Paper forms or records	2 (8.3%)	1 (4.2%)	1 (4.8%)
mHealth or apps	… … ..	… … ..	… … ..
**Provided on-site treatment, yes**	**21 (80.8%)**	**21 (80.8%)**	**14 (53.8%)**
**Systems or methods used**	**n = 21**	**n = 21**	**n = 14**
EHR structured fields	17 (81.0%)	18 (85.7%)	12 (85.7%)
EHR unstructured fields (e.g., progress notes)	10 (47.6%)	11 (52.4%)	6 (42.9%)
Patient Portal	7 (33.3%)	7 (33.3%)	4 (28.6%)
Paper forms or records	2 (9.5%)	3 (14.3%)	1 (7.1%)
mHealth or apps	… … ..	… … ..	… … ..
**Arranged referral to a specialist, yes**	**23 (88.5%)**	**24 (92.3%)**	**23 (88.5%)**
**Systems or methods used**	**n = 23**	**n = 24**	**n = 23**
EHR structured fields	14 (60.9%)	14 (58.3%)	13 (56.5%)
EHR unstructured fields (e.g., progress notes)	19 (82.6%)	21 (87.5%)	18 (78.3%)
Patient Portal	4 (17.4%)	6 (25.0%)	4 (17.4%)
Paper forms or records	6 (26.1%)	8 (33.3%)	5 (21.7%)
mHealth or apps	1 (4.3%)	1 (4.2%)	1 (4.3%)
**Conducted follow-up for uptake of substance use disorder treatment or documentation, yes**	**20 (76.9%)**	**24 (92.3%)**	**23 (88.5%)**
**Systems or methods used**	**n = 20**	**n = 24**	**n = 23**
EHR structured fields	6 (30.0%)	6 (25.0%)	5 (21.7%)
EHR unstructured fields (e.g., progress notes)	19 (95.0%)	23 (95.8%)	23 (100.0%)
Patient Portal	4 (20.0%)	5 (20.8%)	5 (21.7%)
Paper forms or records	2 (10.0%)	3 (12.5%)	5 (21.7%)
mHealth or apps	1 (5.0%)	1 (4.2%)	1 (4.3%)
**Conducted follow-up for other services, yes**	**8 (30.8%)**	**9 (34.6%)**	**4 (15.4%)**
**Systems or methods used**	**n = 8**	**n = 9**	**n = 4**
EHR structured fields	3 (37.5%)	2 (22.2%)	1 (25.0%)
EHR unstructured fields (e.g., progress notes)	7 (87.5%)	8 (88.9%)	4 (100.0%)
Patient Portal	1 (12.5%)	… … ..	1 (25.0%)
Paper forms or records	1 (12.5%)	1 (11.1%)	… … ..
mHealth or apps	… … ..	… … ..	… … ..

In all visit types, both structured and unstructured EHR fields were reported to be commonly used to document SBIRT tasks. The following tasks had especially high proportions of using unstructured EHR fields (e.g., clinical notes) for all visit types given that the task was performed: substance use screen (76.0 percent [19/25] to 88.2 percent [15/17]), treatment option exploration (95.5 percent [21/22] to 96.2 percent [25/26]), provision of brief intervention/counseling (100 percent [24/24 for new and existing patients, 21/21 for patient requiring a referral]), referral to a specialist (78.3 percent [18/23] to 87.5 percent [21/24]), and follow-up of the uptake of SUD treatment (95.0 percent [19/20] to 100 percent [23/23]). Patient portal and paper forms had a low proportion of use, and mHealth was infrequently used.

## Discussion

Substance misuse and addiction is among the top national public health concerns [47]. However, the quality of the SUD preventive service and treatment data in the EHR is understudied to inform the use of the EHR data for health care performance evaluation and research efforts (e.g., patient recruitment and outcome measures for pragmatic trials). In 2018, US Department of Health and Human Services released a five-point strategy report to combat the national opioid crisis aimed at improving (a) SUD prevention, treatment, and recovery services; (b) public health data reporting and collection; (c) pain management treatment services; (d) availability and distribution of overdose-reversing medications; and (e) research related to SUDs and pain [47]. Consequently, the National Institutes of Health’s HEAL Initiative is expected to increase considerably the number of clinical trials of SUD related intervention and treatments in medical settings [39]. The evaluation of such national efforts will require the availability of high-quality SBIRT data in the EHR in order to accurately monitor the progress of national efforts and produce high-quality, real-world findings to impact SUD related treatments. Thus, findings from this study are uniquely important and timely needed to inform the use of the EHR data for SUD related clinical and research activities.

First, the findings suggest a low use of validated tools by providers to screen substance use or assess SUD treatment needs. We used the SBIRT framework, an evidence-based model to provide health care for SUD treatment services, in order to explore whether the EHR would capture key processes and outcome information that would be useful for the purposes for the quality improvement and secondary use of SUD related research [15,25,26,27]. Unlike other chronic diseases that often use biological measures to monitor treatment (e.g., glucose monitoring for diabetes), substance use screening and assessments for SUD treatment needs typically rely on the use of validated instruments to collect patient-reported information [15]. Our results suggest that screening tools and measures for SUD (including treatment needs) are infrequently used by providers [48]. This finding indicates that SUDs are likely to be under-detected or under-intervened by providers, as providers may utilize clinical impressions of substance misuse, or conduct substance use screening irregularly. A prior study found that provider’s clinical impressions of substance misuse underestimated substance misuse problems [49].

Several reasons may be related to a low use of validated tools for SBIRT efforts. There is a general lack of brief tools that are clinically feasible for screening problems from use of multiple substances in a busy clinical setting [50]. The use of validated tools can be constrained by whether they are easily accessible from the EHR and whether they are specified as a ‘required’ field [51]. Providers also may have insufficient knowledge about screening substance use or assessing for SUDs, or be uncomfortable about conducting SBIRT and SUD treatment [48,52,53]. Given that the low use of validated tools also suggest incompleteness or inaccuracy of the SBIRT data in the EHR (e.g., misclassification of SUD status), barriers to utilizing validated tools or providing SBIRT services by providers (e.g., training for SBIRT, EHR functionality to support SBIRT tasks) must be identified and addressed to improve the extent of the SBIRT service data captured in an EHR. For example, institutional support is needed to establish an infrastructure to support SBIRT training and policies for implementing an EHR with embedded decision support tools to guide the performance of SBIRT services. Additionally, the extent of under-detection and under-treatment for SUDs should be carefully evaluated and addressed while the EHR data are utilized for any SUD related clinical and research purpose.

Another important finding concerns the common use of unstructured EHR fields (e.g., clinical notes) to document SBIRT tasks. It appears that clinical tasks related to substance use screening, treatment option exploration, provision of brief intervention, referral to a specialist, and follow-up of the uptake of SUD treatment are likely be recorded in the unstructured EHR fields (e.g., clinical notes), while assessment of SUDs and provision of on-site SUD treatment tended to be documented in the structured fields. These findings may be related to design features of an EHR. In particular, SUD treatments have traditionally been separated from the mainstream medical settings. Providers in general medical settings may be less-familiar with how to document SBIRT information. The existing EHR also may not include an adequate number of structured fields to record SBIRT services. On the other hand, the EHR tends to include structured fields to record diagnosis and prescription for on-site SUD treatment. The overall pattern of the use of an EHR and other methods to document SBIRT tasks is similar across different visit types.

In terms of the implications for the EHR data quality for SUD treatment quality evaluation and research purposes, the use of the information from the structured data fields of the EHR alone will provide incomplete information about patient care profiles and treatment needs for SUDs. Future research is needed to further evaluate the extent of unstructured SBIRT data in the EHR in order to identify strategies to address the challenges of capturing more complete SBIRT data in the structured data fields. To improve the accuracy of the SBIRT service data in the EHR for meeting the quality measures and supporting clinical research, an application of natural language processing and additional technologies is needed to extract information from clinical text into a structured representation and integrate both structured and unstructured EHR data [54,55]. Further, our findings suggest a lower use of patient portal and mHealth in conducting SBIRT services. Future studies may explore whether the sensitive nature of SUD treatment is related to the lower use of patient portal and mHealth for clinical care. Specifically, it would be important to investigate whether concerns about protecting the privacy of SUD treatment (42-CFR part 2: substance abuse confidentiality regulations) or variations in the interpretation of 42-CFR part 2 regulation may affect the documentation of SBIRT services in general and use of patient portal and mHealth in particular for communicating SBIRT services [56,57]. On the other hand, providers are not experts in the EHR functionality. They may not be aware of full EHR data fields for conducting SBIRT services. Future studies may explore whether training of the EHR features and functions may improve the workflow and documentation of provision of SBIRT services [37].

### Limitations and strengths

This study has some limitations. It was not designed for hypothesis-testing. Instead, the exploratory design is appropriate for an under-studied area of research, as it allows quicker collection of new information to inform designs of larger, more expensive studies. Because of the lack of research on the clinical workflow and documentation of SBIRT in the EHR, we are unable to compare our results with prior studies. In this regarding, this study produced timely, clinically new and important findings for informing the need for more research on the quality of SUD data in the EHR. Participants are a convenience sample of providers, and the majority was affiliated with a university. The participant of a given facility was not representative of all providers from the facility, and differences in clinical expertise and the EHR features across facilities could influence the use of an EHR for conducting SBIRT information. This study documents participants’ self-reports of clinical practices that also may be limited by reporting bias. Taken together, the results are likely to reflect greater use of the EHR and documentation of SBIRT data in the EHR than the use for providers from non-academic facilities.

Nonetheless, given the lack of the EHR’s workflow research data specifically for both alcohol and drug use disorders, it is suitable to conduct this descriptive design to explore providers’ clinical workflow for SUD care for hypothesis formation in order to inform subsequent research designs. Although results are exploratory in nature, the study sample included providers from 26 diverse settings across 4 census-defined regions in the United States (South 46.2 percent, West 23.1 percent, Midwest 19.2 percent, Northeast 11.5 percent). This high level of diversity is an important strength.

## Conclusion

This study is a valuable contribution to understand the SBIRT workflow in the EHR outside of unique settings, like VA, after the mandated adoption of the EHR in routine care [36]. The EHR data hold great potential for transforming clinical and safety surveillance research, but the challenges of how to capture complete and accurate EHR data must be identified and addressed to realize its promise [28,58]. We used an SBIRT workflow for preventive services and treatments for SUDs in order to explore whether the quality related process and outcome measures (i.e., SBIRT clinical tasks) would be available and documented in the EHR. As noted from this study, researchers face barriers to using the EHR data for SUD research due to incompleteness of the SBIRT data in the EHR. Our clinical workflow interview data suggest that a low use of validated tools for guiding SBIRT services and common use of unstructured EHR data fields for capturing SBIRT information are important barriers to having complete and accurate SBIRT data in the EHR. Strong support from organizational leaderships, educational interventions to promote the knowledge of conducting SBIRT (including academic detailing or periodic refreshers), and the use of clinical champions may improve implementation of SBIRT services and the collection of SUD treatment data in the EHR [37,53,59].

The overall data, while descriptive, emphasize the need for extra caution in leveraging the EHR data for clinical and research use. Identifying measurable ways to leverage both the structured and unstructured EHR data for research will allow researchers to conduct pragmatic trials, which may cost less than traditional clinical trials and can answer clinical care questions not approachable or feasible through traditional clinical trials [58,60]. For research use, the validity of EHR data should be examined and addressed to improve the overall EHR data quality. Unstructured EHR data should be considered in research efforts to better explain an individual’s history of health and health care utilization. Natural language processing offers a computational means to synthesize unstructured EHR text and clinical notes to identify patterns of diseases and provide quantifications of health care indicators of interest [61].

Finally, the comprehensiveness of SUD treatment data in the EHR is central to the proper application of behavioral health quality measures to meet the federal policies for quality measures [54]. Thus, having structured EHR fields to capture SBIRT tasks is important for ease of extracting clinical data for measuring the meaningful use. There is a clear need to develop provider-friendly EHR systems to collect structured data fields and measures for SBIRT services in order to improve the process of health care quality evaluation and the conduct of pragmatic research for SUDs. The national opioid overdose epidemic in the United States is an important rationale for conducting more research to identify strategies for improving the EHR workflow and documentation of SBIRT data for patients with SUD [62].

**Institutional review board approval:** This work has been approved by the Duke University Health System Institutional Review Board.
